# (2*S*,6*S*)-1-Methyl-2,6-*trans*-distyryl­piperidinium chloride

**DOI:** 10.1107/S1600536809049587

**Published:** 2009-12-09

**Authors:** Guangrong Zheng, Sean Parkin, Linda P. Dwoskin, Peter A. Crooks

**Affiliations:** a Department of Pharmaceutical Sciences, College of Pharmacy, University of Kentucky, Lexington, KY 40536, USA; b Department of Chemistry, University of Kentucky, Lexington, KY 40536, USA

## Abstract

In the crystal structure of the title compound, C_22_H_26_N^+^·Cl^−^, the piperidine ring is in a chair conformation and the two styryl groups are in axial and equatorial positions. The mol­ecule has a hydrogen bond between the NH group and the chloride anion.

## Related literature

The title compound is a *des*-oxygen derivative of epimerized (−)-lobeline (Zheng *et al.*, 2005 ▶).
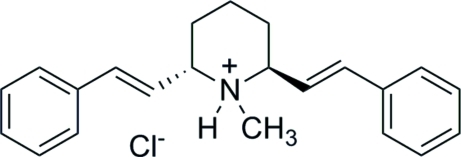


## Experimental

### 

#### Crystal data

C_22_H_26_N^+^·Cl^−^
*M* *_r_* = 339.89
Orthorhombic, 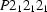

*a* = 9.9355 (4) Å
*b* = 12.3075 (5) Å
*c* = 15.8299 (7) Å
*V* = 1935.70 (14) Å^3^
*Z* = 4
Mo *K*α radiation
μ = 0.20 mm^−1^
*T* = 173 K
0.38 × 0.28 × 0.08 mm

#### Data collection

Nonius KappaCCD diffractometer
Absorption correction: multi-scan (*SCALEPACK*; Otwinowski & Minor, 1997 ▶) *T*_min_ = 0.930, *T*_max_ = 0.984
11921 measured reflections
3416 independent reflections
2957 reflections with *I* > 2σ(*I*)
*R*_int_ = 0.065

#### Refinement

*R*[*F* ^2^ > 2σ(*F* ^2^)] = 0.054
*wR*(*F* ^2^) = 0.091
*S* = 1.11
3416 reflections
218 parameters
H-atom parameters constrained
Δρ_max_ = 0.45 e Å^−3^
Δρ_min_ = −0.26 e Å^−3^
Absolute structure: Flack (1983 ▶), 1457 Friedel pairs
Flack parameter: 0.06 (7)

### 

Data collection: *COLLECT* (Nonius, 1998 ▶); cell refinement: *SCALEPACK* (Otwinowski & Minor, 1997 ▶); data reduction: *DENZO-SMN* (Otwinowski & Minor, 1997 ▶); program(s) used to solve structure: *SHELXS97* (Sheldrick, 2008 ▶); program(s) used to refine structure: *SHELXL97* (Sheldrick, 2008 ▶); molecular graphics: *XP* in Siemens *SHELXTL* (Sheldrick, 2008 ▶); software used to prepare material for publication: *SHELXL97* and local procedures.

## Supplementary Material

Crystal structure: contains datablocks global, I. DOI: 10.1107/S1600536809049587/hg2599sup1.cif

Structure factors: contains datablocks I. DOI: 10.1107/S1600536809049587/hg2599Isup2.hkl

Additional supplementary materials:  crystallographic information; 3D view; checkCIF report

## Figures and Tables

**Table 1:** Hydrogen-bond geometry (Å, °)

*D*—H⋯*A*	*D*—H	H⋯*A*	*D*⋯*A*	*D*—H⋯*A*
N1—H1⋯Cl^i^	0.93	2.10	3.027 (2)	176

Symmetry code: (i) 
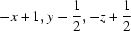
.

**Fig. 1.**
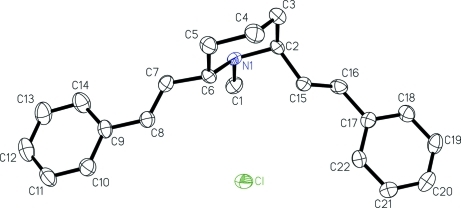
A view of the molecule. Displacement ellipsoids are drawn at the 50% probability level.

## supplementary crystallographic information

### Comment

The title compound is a *des*-oxygen derivative of epimerized (-)-lobeline (Zheng *et al.*, 2005). The molecular structure is illustrated in Fig. 1. The piperidine ring of the molecule is in the chair conformation and the *N*-methyl group is bonded equatorially to the piperidine ring. The N atom has an axial H atom that is hydrogen bonded to the chloride anion (HN..Cl = 3.027 (2) Å). One styryl group is attached equatorially to the piperidine ring and the other styryl group is pseudo-axial, with C15—C2—N1 [111.67 (18)°] and C15—C2—C3 [113.7 (2)°] bond angles slightly different from the ideal 109.5°. The piperidine ring is not mirror symmetric, as indicated by unequal bond lengths and angles (Table 1). The double bond and phenyl ring of the styryl side chain are not coplanar, as evidenced by the C15—C16—C17—C18 and C7—C8—C9—C14 torsion angles, -165.4 (3)° and -169.0 (2)°, respectively.

### Experimental

The title compound was prepared from (-)-lobeline (Zheng *et al.*, 2005). Crystals suitable for X-ray diffraction studies were obtained by slow recrystallization from a solution in methanol and diethyl ether.

### Refinement

H atoms were found in difference Fourier maps and subsequently placed in idealized positions with constrained distances of 0.98 Å (RCH_3_), 0.99 Å (*R*_2_CH_2_), 1.00 Å (*R*_3_CH), 0.95 Å (*R*_2_CH), 0.93 Å (N—H), and with *U*_iso_(H) values set to either 1.2*U*_eq_ or 1.5*U*_eq_ (RCH_3_) of the attached atom.

### Crystal data

**Table:**

C_22_H_26_N^+^·Cl^−^	*F*(000) = 728
*M**_r_* = 339.89	*D*_x_ = 1.166 Mg m^−^^3^
Orthorhombic, *P*2_1_2_1_2_1_	Mo *K*α radiation, λ = 0.71073 Å
Hall symbol: P 2ac 2ab	Cell parameters from 21849 reflections
*a* = 9.9355 (4) Å	θ = 1.0–27.5°
*b* = 12.3075 (5) Å	µ = 0.20 mm^−^^1^
*c* = 15.8299 (7) Å	*T* = 173 K
*V* = 1935.70 (14) Å^3^	Irregular plates, colourless
*Z* = 4	0.38 × 0.28 × 0.08 mm

### Data collection

**Table:**

Nonius KappaCCD diffractometer	3416 independent reflections
Radiation source: fine-focus sealed tube	2957 reflections with *I* > 2σ(*I*)
graphite	*R*_int_ = 0.065
Detector resolution: 18 pixels mm^-1^	θ_max_ = 25.0°, θ_min_ = 2.1°
ω scans at fixed χ = 55°	*h* = −11→11
Absorption correction: multi-scan (*SCALEPACK*; Otwinowski & Minor, 1997)	*k* = −14→14
*T*_min_ = 0.930, *T*_max_ = 0.984	*l* = −18→18
11921 measured reflections	

### Refinement

**Table:**

Refinement on *F*^2^	Secondary atom site location: difference Fourier map
Least-squares matrix: full	Hydrogen site location: inferred from neighbouring sites
*R*[*F*^2^ > 2σ(*F*^2^)] = 0.054	H-atom parameters constrained
*wR*(*F*^2^) = 0.091	*w* = 1/[σ^2^(*F*_o_^2^) + (0.0332*P*)^2^ + 0.1721*P*] where *P* = (*F*_o_^2^ + 2*F*_c_^2^)/3
*S* = 1.11	(Δ/σ)_max_ = 0.001
3416 reflections	Δρ_max_ = 0.45 e Å^−^^3^
218 parameters	Δρ_min_ = −0.26 e Å^−^^3^
0 restraints	Absolute structure: Flack (1983), 1457 Friedel pairs
Primary atom site location: structure-invariant direct methods	Flack parameter: 0.06 (7)

### Special details

**Table:**

Geometry. All e.s.d.'s (except the e.s.d. in the dihedral angle between two l.s. planes) are estimated using the full covariance matrix. The cell e.s.d.'s are taken into account individually in the estimation of e.s.d.'s in distances, angles and torsion angles; correlations between e.s.d.'s in cell parameters are only used when they are defined by crystal symmetry. An approximate (isotropic) treatment of cell e.s.d.'s is used for estimating e.s.d.'s involving l.s. planes.
Refinement. Refinement of *F*^2^ against ALL reflections. The weighted *R*-factor *wR* and goodness of fit *S* are based on *F*^2^, conventional *R*-factors *R* are based on *F*, with *F* set to zero for negative *F*^2^. The threshold expression of *F*^2^ > σ(*F*^2^) is used only for calculating *R*-factors(gt) *etc*. and is not relevant to the choice of reflections for refinement. *R*-factors based on *F*^2^ are statistically about twice as large as those based on *F*, and *R*- factors based on ALL data will be even larger.

### Fractional atomic coordinates and isotropic or equivalent isotropic displacement parameters (Å^2^)

**Table:**

	*x*	*y*	*z*	*U*_iso_*/*U*_eq_	
Cl	0.49125 (6)	1.13417 (4)	0.35616 (4)	0.03556 (19)	
N1	0.44499 (17)	0.82481 (15)	0.25784 (13)	0.0275 (5)	
H1	0.4661	0.7643	0.2253	0.033*	
C1	0.4017 (3)	0.9121 (2)	0.19752 (16)	0.0358 (7)	
H1A	0.3144	0.8928	0.1729	0.054*	
H1B	0.4687	0.9192	0.1524	0.054*	
H1C	0.3938	0.9812	0.2278	0.054*	
C2	0.3304 (2)	0.79138 (19)	0.31510 (17)	0.0305 (6)	
H2	0.2579	0.7607	0.2783	0.037*	
C3	0.3774 (3)	0.7007 (2)	0.37336 (17)	0.0372 (7)	
H3A	0.3035	0.6812	0.4125	0.045*	
H3B	0.3991	0.6356	0.3392	0.045*	
C4	0.5004 (3)	0.7336 (2)	0.42414 (16)	0.0395 (7)	
H4A	0.4780	0.7958	0.4612	0.047*	
H4B	0.5298	0.6724	0.4602	0.047*	
C5	0.6127 (2)	0.76520 (19)	0.36400 (16)	0.0331 (6)	
H5A	0.6390	0.7008	0.3304	0.040*	
H5B	0.6920	0.7884	0.3972	0.040*	
C6	0.5726 (2)	0.85631 (19)	0.30435 (15)	0.0272 (6)	
H6	0.5545	0.9232	0.3383	0.033*	
C7	0.6841 (2)	0.87992 (19)	0.24306 (15)	0.0292 (6)	
H7	0.7039	0.8283	0.2002	0.035*	
C8	0.7558 (2)	0.9709 (2)	0.24688 (15)	0.0283 (6)	
H8	0.7260	1.0241	0.2861	0.034*	
C9	0.8759 (2)	0.9986 (2)	0.19738 (15)	0.0281 (6)	
C10	0.9516 (2)	1.0897 (2)	0.22012 (17)	0.0342 (7)	
H10	0.9237	1.1327	0.2667	0.041*	
C11	1.0659 (2)	1.1182 (2)	0.17623 (18)	0.0391 (7)	
H11	1.1164	1.1800	0.1932	0.047*	
C12	1.1074 (3)	1.0578 (2)	0.10788 (17)	0.0444 (8)	
H12	1.1859	1.0780	0.0775	0.053*	
C13	1.0347 (3)	0.9682 (2)	0.08396 (17)	0.0460 (8)	
H13	1.0629	0.9266	0.0366	0.055*	
C14	0.9202 (3)	0.9378 (2)	0.12838 (17)	0.0419 (7)	
H14	0.8715	0.8750	0.1116	0.050*	
C15	0.2714 (2)	0.88730 (18)	0.36100 (16)	0.0278 (6)	
H15	0.3300	0.9378	0.3875	0.033*	
C16	0.1402 (2)	0.90289 (19)	0.36523 (17)	0.0307 (6)	
H16	0.0856	0.8520	0.3359	0.037*	
C17	0.0685 (2)	0.9906 (2)	0.41046 (14)	0.0257 (6)	
C18	−0.0696 (2)	0.9803 (2)	0.42416 (15)	0.0312 (6)	
H18	−0.1147	0.9167	0.4054	0.037*	
C19	−0.1417 (3)	1.0608 (2)	0.46452 (16)	0.0383 (7)	
H19	−0.2359	1.0527	0.4729	0.046*	
C20	−0.0774 (3)	1.1519 (2)	0.49227 (17)	0.0390 (7)	
H20	−0.1269	1.2071	0.5204	0.047*	
C21	0.0595 (3)	1.1644 (2)	0.47978 (16)	0.0342 (7)	
H21	0.1037	1.2278	0.4998	0.041*	
C22	0.1322 (3)	1.0848 (2)	0.43814 (15)	0.0290 (6)	
H22	0.2258	1.0945	0.4284	0.035*	

### Atomic displacement parameters (Å^2^)

**Table:**

	*U*^11^	*U*^22^	*U*^33^	*U*^12^	*U*^13^	*U*^23^
Cl	0.0428 (4)	0.0231 (3)	0.0408 (4)	−0.0033 (3)	0.0079 (3)	−0.0019 (3)
N1	0.0250 (11)	0.0208 (11)	0.0369 (12)	0.0017 (9)	0.0023 (10)	−0.0077 (10)
C1	0.0337 (16)	0.0357 (15)	0.0379 (16)	0.0067 (13)	−0.0034 (13)	−0.0001 (14)
C2	0.0190 (14)	0.0247 (14)	0.0478 (16)	−0.0070 (11)	0.0024 (13)	−0.0055 (13)
C3	0.0334 (16)	0.0283 (15)	0.0500 (18)	−0.0045 (12)	0.0089 (14)	0.0034 (14)
C4	0.0414 (16)	0.0329 (14)	0.0442 (17)	0.0019 (15)	0.0055 (16)	0.0147 (13)
C5	0.0243 (14)	0.0306 (15)	0.0445 (17)	0.0002 (12)	−0.0047 (13)	0.0062 (14)
C6	0.0194 (13)	0.0244 (14)	0.0377 (15)	−0.0057 (12)	−0.0040 (12)	−0.0056 (13)
C7	0.0256 (13)	0.0319 (15)	0.0301 (15)	0.0050 (13)	−0.0009 (12)	−0.0023 (13)
C8	0.0236 (14)	0.0294 (15)	0.0319 (15)	0.0007 (12)	−0.0023 (12)	−0.0012 (13)
C9	0.0218 (14)	0.0360 (16)	0.0264 (14)	0.0004 (12)	−0.0029 (12)	0.0050 (13)
C10	0.0288 (15)	0.0325 (15)	0.0412 (16)	0.0026 (12)	−0.0026 (13)	0.0033 (13)
C11	0.0294 (15)	0.0356 (17)	0.0523 (19)	−0.0086 (13)	−0.0001 (14)	0.0098 (16)
C12	0.0300 (16)	0.061 (2)	0.0427 (18)	−0.0069 (16)	0.0042 (14)	0.0142 (16)
C13	0.0379 (18)	0.066 (2)	0.0339 (17)	−0.0039 (16)	0.0074 (14)	−0.0072 (15)
C14	0.0347 (16)	0.0530 (18)	0.0380 (18)	−0.0100 (14)	−0.0044 (14)	−0.0036 (16)
C15	0.0241 (14)	0.0235 (14)	0.0359 (15)	−0.0025 (11)	0.0007 (12)	−0.0038 (12)
C16	0.0280 (15)	0.0262 (14)	0.0379 (15)	−0.0046 (11)	−0.0065 (13)	−0.0035 (13)
C17	0.0231 (14)	0.0276 (15)	0.0265 (14)	0.0052 (12)	−0.0040 (11)	0.0030 (12)
C18	0.0238 (15)	0.0347 (16)	0.0350 (15)	−0.0033 (13)	−0.0051 (12)	−0.0003 (13)
C19	0.0237 (15)	0.0502 (19)	0.0410 (18)	0.0055 (14)	0.0035 (13)	−0.0038 (15)
C20	0.0382 (18)	0.0449 (19)	0.0338 (16)	0.0115 (15)	0.0034 (13)	−0.0065 (15)
C21	0.0378 (17)	0.0297 (17)	0.0352 (16)	−0.0010 (13)	0.0011 (13)	−0.0052 (13)
C22	0.0224 (14)	0.0335 (15)	0.0311 (15)	−0.0016 (12)	0.0019 (12)	0.0009 (13)

### Geometric parameters (Å, °)

**Table:**

N1—C1	1.500 (3)	C9—C10	1.397 (3)
N1—C2	1.512 (3)	C10—C11	1.377 (3)
N1—C6	1.517 (3)	C10—H10	0.9500
N1—H1	0.9300	C11—C12	1.376 (3)
C1—H1A	0.9800	C11—H11	0.9500
C1—H1B	0.9800	C12—C13	1.371 (4)
C1—H1C	0.9800	C12—H12	0.9500
C2—C15	1.505 (3)	C13—C14	1.389 (3)
C2—C3	1.522 (3)	C13—H13	0.9500
C2—H2	1.0000	C14—H14	0.9500
C3—C4	1.518 (3)	C15—C16	1.320 (3)
C3—H3A	0.9900	C15—H15	0.9500
C3—H3B	0.9900	C16—C17	1.478 (3)
C4—C5	1.517 (3)	C16—H16	0.9500
C4—H4A	0.9900	C17—C22	1.392 (3)
C4—H4B	0.9900	C17—C18	1.394 (3)
C5—C6	1.519 (3)	C18—C19	1.380 (3)
C5—H5A	0.9900	C18—H18	0.9500
C5—H5B	0.9900	C19—C20	1.363 (4)
C6—C7	1.501 (3)	C19—H19	0.9500
C6—H6	1.0000	C20—C21	1.383 (3)
C7—C8	1.329 (3)	C20—H20	0.9500
C7—H7	0.9500	C21—C22	1.384 (3)
C8—C9	1.468 (3)	C21—H21	0.9500
C8—H8	0.9500	C22—H22	0.9500
C9—C14	1.395 (3)		
			
C1—N1—C2	111.13 (18)	C7—C8—C9	127.4 (2)
C1—N1—C6	111.45 (18)	C7—C8—H8	116.3
C2—N1—C6	114.09 (19)	C9—C8—H8	116.3
C1—N1—H1	106.5	C14—C9—C10	117.5 (2)
C2—N1—H1	106.5	C14—C9—C8	123.4 (2)
C6—N1—H1	106.5	C10—C9—C8	119.1 (2)
N1—C1—H1A	109.5	C11—C10—C9	121.2 (3)
N1—C1—H1B	109.5	C11—C10—H10	119.4
H1A—C1—H1B	109.5	C9—C10—H10	119.4
N1—C1—H1C	109.5	C12—C11—C10	120.4 (3)
H1A—C1—H1C	109.5	C12—C11—H11	119.8
H1B—C1—H1C	109.5	C10—C11—H11	119.8
C15—C2—N1	111.67 (18)	C13—C12—C11	119.6 (3)
C15—C2—C3	113.7 (2)	C13—C12—H12	120.2
N1—C2—C3	109.39 (19)	C11—C12—H12	120.2
C15—C2—H2	107.2	C12—C13—C14	120.6 (3)
N1—C2—H2	107.2	C12—C13—H13	119.7
C3—C2—H2	107.2	C14—C13—H13	119.7
C4—C3—C2	111.81 (19)	C13—C14—C9	120.7 (3)
C4—C3—H3A	109.3	C13—C14—H14	119.7
C2—C3—H3A	109.3	C9—C14—H14	119.7
C4—C3—H3B	109.3	C16—C15—C2	121.6 (2)
C2—C3—H3B	109.3	C16—C15—H15	119.2
H3A—C3—H3B	107.9	C2—C15—H15	119.2
C5—C4—C3	109.2 (2)	C15—C16—C17	127.4 (2)
C5—C4—H4A	109.8	C15—C16—H16	116.3
C3—C4—H4A	109.8	C17—C16—H16	116.3
C5—C4—H4B	109.8	C22—C17—C18	118.3 (2)
C3—C4—H4B	109.8	C22—C17—C16	122.8 (2)
H4A—C4—H4B	108.3	C18—C17—C16	118.9 (2)
C4—C5—C6	112.74 (19)	C19—C18—C17	121.2 (3)
C4—C5—H5A	109.0	C19—C18—H18	119.4
C6—C5—H5A	109.0	C17—C18—H18	119.4
C4—C5—H5B	109.0	C20—C19—C18	119.7 (2)
C6—C5—H5B	109.0	C20—C19—H19	120.1
H5A—C5—H5B	107.8	C18—C19—H19	120.1
C7—C6—N1	110.65 (18)	C19—C20—C21	120.5 (3)
C7—C6—C5	110.60 (19)	C19—C20—H20	119.8
N1—C6—C5	109.39 (19)	C21—C20—H20	119.8
C7—C6—H6	108.7	C20—C21—C22	120.2 (3)
N1—C6—H6	108.7	C20—C21—H21	119.9
C5—C6—H6	108.7	C22—C21—H21	119.9
C8—C7—C6	122.0 (2)	C21—C22—C17	120.1 (2)
C8—C7—H7	119.0	C21—C22—H22	119.9
C6—C7—H7	119.0	C17—C22—H22	119.9
			
C1—N1—C2—C15	−54.9 (3)	C8—C9—C10—C11	179.3 (2)
C6—N1—C2—C15	72.2 (2)	C9—C10—C11—C12	0.7 (4)
C1—N1—C2—C3	178.35 (18)	C10—C11—C12—C13	−0.4 (4)
C6—N1—C2—C3	−54.6 (3)	C11—C12—C13—C14	−0.4 (4)
C15—C2—C3—C4	−69.2 (3)	C12—C13—C14—C9	0.9 (4)
N1—C2—C3—C4	56.4 (3)	C10—C9—C14—C13	−0.6 (4)
C2—C3—C4—C5	−58.0 (3)	C8—C9—C14—C13	180.0 (2)
C3—C4—C5—C6	57.4 (3)	N1—C2—C15—C16	133.2 (3)
C1—N1—C6—C7	−57.5 (2)	C3—C2—C15—C16	−102.5 (3)
C2—N1—C6—C7	175.62 (18)	C2—C15—C16—C17	177.6 (2)
C1—N1—C6—C5	−179.60 (19)	C15—C16—C17—C22	16.4 (4)
C2—N1—C6—C5	53.5 (2)	C15—C16—C17—C18	−165.4 (3)
C4—C5—C6—C7	−176.7 (2)	C22—C17—C18—C19	−0.4 (4)
C4—C5—C6—N1	−54.6 (3)	C16—C17—C18—C19	−178.7 (2)
N1—C6—C7—C8	129.0 (2)	C17—C18—C19—C20	−0.5 (4)
C5—C6—C7—C8	−109.6 (3)	C18—C19—C20—C21	0.4 (4)
C6—C7—C8—C9	172.7 (2)	C19—C20—C21—C22	0.6 (4)
C7—C8—C9—C14	10.4 (4)	C20—C21—C22—C17	−1.5 (4)
C7—C8—C9—C10	−169.0 (2)	C18—C17—C22—C21	1.4 (4)
C14—C9—C10—C11	−0.2 (3)	C16—C17—C22—C21	179.7 (2)

### Hydrogen-bond geometry (Å, °)

**Table:**

*D*—H···*A*	*D*—H	H···*A*	*D*···*A*	*D*—H···*A*
N1—H1···Cl^i^	0.93	2.10	3.027 (2)	176

Symmetry codes: (i) −*x*+1, *y*−1/2, −*z*+1/2.
